# Shared decision-making with older people on TReatment Escalation planning for Acute deterioration in the emergency Medical Setting: a qualitative study of Clinicians’ perspectives (STREAMS-C)

**DOI:** 10.1093/ageing/afae204

**Published:** 2024-09-25

**Authors:** Bronwen E Warner, Mary Wells, Cecilia Vindrola-Padros, Stephen J Brett

**Affiliations:** Division of Anaesthetics, Pain Management and Intensive Care, Department of Surgery and Cancer, Imperial College London, London, SW7 2AZ, UK; Department of Surgery and Cancer, Imperial College London, London, SW7 2AZ, UK; Directorate of Nursing, Imperial College Healthcare NHS Trust, London, W6 8RF, UK; Department of Targeted Intervention, University College London, London, WC1E 6BT, UK; Division of Anaesthetics, Pain Management and Intensive Care, Department of Surgery and Cancer, Imperial College London, London, SW7 2AZ, UK; Department of Intensive Care Medicine, Imperial College Healthcare NHS Trust, London, W120HS, UK

**Keywords:** shared decision-making, treatment escalation planning, cardiopulmonary resuscitation, doctor–patient relationship, qualitative research, older people

## Abstract

**Background:**

Shared decision-making (SDM) is increasingly expected in healthcare systems prioritising patient autonomy. Treatment escalation plans (TEPs) outline contingency for medical intervention in the event of patient deterioration. This study aimed to understand clinicians’ perspectives on SDM in TEP for older patients in the acute medical setting.

**Methods:**

This was a qualitative study following a constructivist approach. Semistructured interviews with vignettes were conducted with 26 consultant and registrar doctors working in emergency medicine, general internal medicine, intensive care medicine and palliative care medicine. Reflexive thematic analysis was performed.

**Results:**

There were three themes: ‘An unequal partnership’, ‘Options without equipoise’ and ‘Decisions with shared understanding’. Clinicians’ expertise in synthesising complex, uncertain clinical information was contrasted with perceived patient unfamiliarity with future health planning and medical intervention. There was a strong sense of morality underpinning decision-making and little equipoise about appropriate TEP decisions. Communication around the TEP was important, and clinicians sought control over the high-stakes decision whilst avoiding conflict and achieving shared understanding.

**Conclusions:**

Clinicians take responsibility for securing a ‘good’ TEP decision for older patients in the acute medical setting. They synthesise clinical data with implicit ethical reasoning according to their professional predictions of qualitative and quantitative success following medical intervention. SDM is seldom considered a priority for this context. Nonetheless, avoidance of conflict, preserving the clinical relationship and shared understanding with the patient and family are important.

## Key Points

- Shared decision-making (SDM) is widely cited in UK guidance including in treatment escalation planning (TEP). However, a recent systematic review shows that TEP decisions are dominated by clinicians [ 1]. This study aimed to understand clinicians’ perspectives on the role of SDM in TEP in the acute medical setting for older patients with capacity to make decisions about TEP.
- This qualitative interview study with UK physicians demonstrates that TEP decisions are complex, intuitive and ethically motivated, such that clinicians feel responsibility to ensure ‘good’ decisions. Whilst patient understanding and agreement are important, SDM is seldom considered pertinent to appropriate TEP decision-making.
- Guidance emphasises patient involvement in healthcare decision-making, but UK clinicians are expected to determine subjectively a ‘realistic’ chance of cardiopulmonary resuscitation success and are not required to provide treatment escalation they consider inappropriate. This study increases understanding of clinicians’ approach to formulating what they view to be appropriate TEP decisions. For clinicians conducting TEP conversations in their own practice, this study presents a nuanced analysis of participants’ views alongside description of current guidelines, which may provoke reflection and learning. For policy makers, our study adds perspective to inform practicable guidelines in the context of recurrent legal deliberation on this high-stakes and emotive topic.

## Introduction

Shared decision-making (SDM) is increasingly expected in healthcare [2–6], including during treatment escalation planning (TEP) [7]. The population is ageing [8, 9] and older people, who may be frail or have multiple interacting comorbidities [10, 11], are consequently admitted to hospital in high numbers [12]. Understanding nuances of SDM around TEP for older patients is relevant for clinicians, patients and policy makers.

‘Shared decision-making’ in medicine means incorporating clinician evidence with informed patient preferences [2]. SDM reflects wider societal expectations in Western contexts for individual autonomy [13–17] and may increase patient satisfaction [5, 18]. It is now endorsed across almost all clinical settings in the UK [5] and may be particularly important for older people to establish an overall focus of care when there are competing priorities [19–21].

TEPs are a component of advanced care planning (ACP), which encompasses holistic decision-making around deteriorating health. TEPs outline a contingency for extent of medical intervention such as organ support and cardiopulmonary resuscitation (CPR), recognising that treatment may not prolong life or achieve a quality of life acceptable to the patient [22]. TEPs are ideally considered whilst a patient has capacity to contribute to decisions about their care, so that views are known if capacity is lost [23].

There exists some tension around the role of SDM in TEP. Current guidelines emphasise the importance of patient involvement in decision-making around CPR, especially where there is a ‘realistic chance of success’ for a ‘sustained period’ and benefits and burdens of treatment need to be considered [24]. However, clinicians are generally not required to offer treatment they judge would be nonbeneficial [5, 25], and a recent systematic review suggested that clinicians do not prioritise SDM in TEP [1]. The role of patients in decision-making generally remains complex: there is increasing emphasis on SDM and patient-centred care [3, 26, 27] and acceptance that capacitous patients can make ‘unwise decisions’; [28] but consensus that clinicians are usually not required to provide treatment they consider would not ‘serve the patient’s needs’ [3]. Patient involvement in healthcare decisions is increasingly subject to legal deliberation [29, 30] and debate in the popular press [31].

Understanding stakeholder perspectives may be helpful in informing practicable standards on when and how SDM should be employed in TEP. This study aimed to answer the research question: what are clinicians’ perspectives on the role of SDM in TEP in the acute medical inpatient setting for older patients with capacity?

## Methods

### Study design

STREAMS-C was a qualitative study of clinicians in an acute generalist hospital setting in Northwest London.

Approval was obtained from the Health Research Authority ref 22/HRA/4387. Patient and public involvement has informed the programme of research of which this study is part.

Participants were consultant and registrar-grade clinicians (independent TEP decision-makers with >4 years clinical experience) working in emergency medicine (EM), general internal medicine (GIM), intensive care medicine (ICM) and palliative care. These specialties routinely encounter older comorbid patients presenting as unplanned acute medical admissions, and it was anticipated that they would bring a range of perspectives. Participants were recruited from three hospitals at one large National Health Service (NHS) Trust.

There was purposive stratified sampling to capture a balance of specialties and seniority. Potential participants were approached via email or in person by the research team and departmental leads were invited to disseminate the invitation via email. The approximate anticipated number of participants was informed by information power, incorporating breadth of the research question, relevance of the sample and complexity of the topic; [32] recruitment continued until data saturation was approached, identified by idea repetition during the final three interviews [33].

### Participant interviews and data collection

Informed, written consent was obtained in advance and verbal consent recorded again at the beginning of each interview.

Semi-structured interviews were conducted using topic guides supported by short vignettes to prompt discussion [34]. Further details are available in Appendix 1.

Interviews were conducted November 2022–February 2023. They lasted an average of 61 minutes, with a range of 35–79 minutes. Five took place in person and the remainder via video conferencing. Interviews were audio-recorded and then transcribed by an external transcription company (Way With Words, London, UK. https://waywithwords.net/).

### Reflexivity

The interviewer B.E.W. is a physician undertaking a PhD. The wider team comprised an Intensive Care consultant with academic interest in Intensive Care Unit (ICU) decision-making (S.J.B.), professor of cancer nursing with experience in qualitative methods (M.W.) and a medical anthropologist (C.V.P.). S.J.B. and B.E.W. work clinically with some of the participants. The team was aware that their clinical perspectives of TEP decision-making could inform design and analysis where views most congruent with their own might be most easily received, and that colleague dynamics could influence interviews. B.E.W. kept a research diary and debriefed with the wider team to maintain openness and critical reflection. A detailed reflexive account has been published separately [35].

### Data analysis

Thematic analysis (TA) seeks to identify patterns of meaning as themes across a data set through coding [36]. Reflexive TA (rTA) is a subtype of TA emphasising critical reflection by the researcher [37]. This study recognised a relativist ontology, reflecting SDM as a social construct, and a constructivist epistemology wherein the participants actively interpret and construct meaning from their lived experience. B.E.W. developed the rTA inductively according to the six-step approach in consultation with the wider research team. Credibility and dependability are demonstrated through a detailed description of the analytic process, reflexivity and research team debriefing (further details in Appendix 2) [37].

Findings are reported in accordance with the consolidated criteria for reporting qualitative research (Appendix 3) [38].

## Results

There were 26 clinicians interviewed, comprising 6 EM, 9 GIM, 7 ICM and 4 palliative care, of which 15 were consultants and 11 registrar level ST3 and above. There were 11 female and 15 male participants.

Findings are presented under the three themes of ‘an unequal partnership’, ‘options without equipoise’ and ‘decisions with shared understanding’. Themes, subthemes and illustrative quotes are included in Table 1. A detailed description of findings in context is in Appendix 4.

**Table 1:** Themes, subthemes and illustrative quotes. EM = emergency medicine, ICM = intensive care medicine, GIM = general internal medicine

Theme/subtheme	Quote	Participant
AN UNEQUAL PARTNERSHIP The factors informing a TEP decision are so complex and unfamiliar that patients and clinicians are not a meaningful partnership to make decisions		
Clinicians synthesise and interpret complex information amidst uncertainty to make TEP decisions	Try and get as much background information for an individual as possible, as well as just the ambulance sheet and the history whenever they arrive, and you’re treating them somewhere in A&E. But maybe look back and see what their past medical history is, how many hospital admissions they’ve had recently, what their social background is, where they’re living. Are they independent at home? Are they dependent in a nursing home? Put that all together.	GIM SpR
	The fact that he has commode and Zimmer that come up there. Those two things, for me, mean that’s it for him, he’s not going to be leaving intensive care or leaving the hospital. I think when there are certain words that will come up or certain things that come from the history, both comorbidity and social history, that’s when I know that that’s when the grey area turns to very much black and white.	ICM Consultant
	So, I think, for me, I think, we’re definitely getting into the territory of would I get him back, probably not. But actually, even if I did, he’s going to be really unwell. Potentially after that his baseline is going to be very different. And actually, his prognosis after all of that would probably be really short anyway.	Palliative Care Consultant
	I think, if he were to arrest, his heart is already poorly with ischaemic heart disease. It’s going to be really hard to resuscitate or get him back. He’s got a history of atrial fibrillation, even though he’s on direct oral anticoagulant, he might still have clots that might give him constant strokes. Or it could go the other way, he might end up bleeding because he’s on oral anticoagulant. If he did not arrest and he just deteriorates from respiratory point of view, he’s got a background of asthma, he’s an ex-smoker. He would probably have a baseline, loss in his respiratory reserve. And it’s never a single organ failure when the heart or the lungs are involved, they bring the other organ along with them. If I end up tubing him first, then I would not manage to extubate him because his dependence on the ventilation would be quite significant. And once he is on positive pressure ventilation that again can cause a heart problem. It can cause varied pressure changes and stuff. Overall, I think if he were to deteriorate he could end up with multi-organ failure.	ICM SpR
	I mean he’s got a urine tract infection. So, if he needs treatment escalation, the chances are probably going to be (he would need) vasopressors	ICM Consultant
	I think we end up, rather than using scoring systems, it’s often done on a gut feel as to whether or not knowing what the journey lies ahead for the majority of patients. Do I think this patient should go or not?	ICM Consultant
	Yes, well there is a reason that we’ve had all the medical training and experience that we have because we see that. We see patients that go to ITU that do well or don’t do well.	GIM SpR
	The truth is yes, usually, you kind of know, at the very least, there are ones where you would say almost certainly this person should be resuscitated, they’re reasonably fit and well. So, those are reasonably straightforward unless the person just happens to have a very strong feeling about something, which you’re going to want to explore as well to make sure it’s not completely based on nonsense. And then there’s a bunch of very obviously would not be resuscitated nowhere near fit enough to survive a resuscitation, ITU admission and so on.	GIM Consultant
Balancing acceptable outcomes within the limitations of treatment possibilities is unfamiliar to the general public	And I don’t think necessarily we live in a time where people talk about death as frankly as they used to	EM Consultant
	They’re a bit shocked and it derails their consultation in a slightly negative way	GIM Consultant
	It’s easier if they’ve done it already, if they’ve broached the subject already. But if they’ve never heard the word ‘resuscitation’ before, then I think it puts a barrier between us and the patient	GIM Consultant
	And they say, well, make me walk, doctor. And then you say, sure, but you’ve not walked for ten years, and you actually don’t have any legs. And that is. . . There’s such a mismatch into their expectations. And same with the family, sometimes, they say, well, we need you to get him better, we need you to get him. . . A year ago he was doing his own shopping and now he’s bedbound, and on a. . . And needs to be fed. We need you to get him back to how he was a year ago, and I would say that’s a mismatch in the goal of care that we can’t offer.	GIM Consultant
	Human nature is we fight to survive so being alive is better than not if you speak to someone on the street about it. So, they don’t really necessarily know what a life of being wheelchair bound or PEG fed or not being able to walk again or not being able to care for yourself and going into a care home is actually really like, especially if you haven’t had to see elderly relatives go through that.	GIM SpR
	We make these decisions for people in the middle of the night, who haven’t met the patient. But actually, for someone like him, that could change. He might suddenly go, actually, now I’m facing death from hypotension and organ failure. Actually I might want some vasopressors, even if he’d said he didn’t before.	ICM Consultant
	From my experience I think he would understand. And he would possibly be on the same grounds as we are because it looks like he’s been through a lot. He’s had a bypass. He can’t do things independently. He’s on a package of care. I think he’ll be on the same page as me.	ICM SpR
	Probably it’s quite vague to say we’ll do everything to support you, which, I don’t know, maybe is vague, but I don’t if it means anything for someone who’s not medical to say, we’ll give you inotropes . . . and put a big line. . . in your neck	GIM Consultant
	You can’t, the whole point is that they have to give me some level of trust. They’ve come to you, you’ve not dragged them, I’ve not dragged them from the street, and said, I’m going to treat you, you’ve come to me. For this to work there’s got to be a little bit of trust there	EM SpR
OPTIONS WITHOUT EQUIPOISE There are right and wrong TEP decisions, for which doctors take responsibility		
There are ethically appropriate TEP decisions	I think you’re always going to put your own idea on what’s a good quality of life. Having seen it and having seen patients come in, they’re so dependent, and I think then, yes.	GIM Consultant
	what sort of chance for any individual is small enough that it’s not worth doing. If you said to most people, if your heart stopped there’s a 1% chance we could get you back to where you were. I think a lot of people might want that. I think this is going to be less than 1%, isn’t it? At that level, I’m comfortable that he’s not going to survive it. I wouldn’t even say the chances are that you won’t, I’ll say, you won’t survive this. I’ve never come across someone who has survived this.	ICM Consultant GIM Consultant
	There’s something very ugly about trying to provide, I mean make a tube for a 92 year old gentleman and chest compressions, it’s just . . . wrong.	EM SpR
	There’s a junior doctor team and it’s traumatic for them and the nurses, and then it’s utilising resources poorly as well	GIM Consultant
	My philosophy to this is I know we can do lots of things, but it doesn’t mean that’s right for the individual in front of you.	GIM SpR
	We’re not obliged to do treatments that we think are going to be ineffective.	ICM Consultant
	That’s not to say that objectively, there’s a right decision. It’s obviously a subjective thing.	GIM Consultant
	I think there is (a right decision). Hopefully there is, otherwise we’re all just chasing our tails, trying to figure out what’s the right of three identical outcomes.	ICM SpR
	And I guess, medically you have your boundaries about things before you go into the conversation, if that makes sense. So, you know what medically doesn’t make sense and what you’re not putting on the table.	Palliative Care Consultant
	So I guess, again, that goes back to our guiding principle from the GMC, and the ethics is you can refuse but you can’t demand. So if they refuse then that answers that. And if they state they don’t want to be resuscitated, then that’s the most important thing, and we need to know that.	GIM Consultant
Doctors shoulder and professional responsibility for the TEP decision	The one thing that I wanted to say to you, I think, just from the perspective of consultants. I don’t think consultants ever set out to do these things badly. Ever. I think, there is a combination of lack of education and training maybe. Lack of communication skills. Lack of confidence. But at the end of the day, I don’t think I’ve ever met a clinician that doesn’t want the best outcome for their patients.	Palliative Care Consultant
	It’s the same way that, sure, I can make a shared decision with my mortgage lender, but ultimately I don’t know what I’m doing, and they will be giving me the money.	GIM Consultant
	it does still reflect on you and you will still get blamed as looking after that patient if that patient is resuscitated and then intubated. And the ITU team look at the patient and the comorbidity state, and go, this patient actually shouldn’t have been resuscitated. So, you’ll still have to answer for that decision.	GIM Consultant
	I think it’s just the different experiences really, and different, maybe, religious beliefs, personal beliefs, social beliefs. I think all of those have an effect, whether they think someone will do well in ITU or not.	ICM Consultant
	I’ve had so many late nights, staying late at work, shoving the NIV machine on the patient and them dying, and nobody even know they were dead because the NIV was making their chest move.	GIM Consultant
	I’ve never had, touch wood, yet, a discussion with a colleague that we’ve completely disagreed on the big goal, the big outcome and it might be how we get there or levels that we do get there and, generally, it’s resolvable with a few points of back and forth.	EM Consultant
DECISIONS WITH SHARED UNDERSTANDING The TEP conversation should ideally achieve patient involvement, understanding and agreement.		
Achieving patient understanding and agreement with the TEP decision is important	I think most experienced doctors will have a little spiel that they pretty much always say, and then they adapt slightly for different patients.	GIM Consultant
	Is it a shared decision as long as we reach the decision that we want I guess is my thought about that because I think we may end up with a lot of people saying I want to be for everything and that isn’t right based on their presentation.	GIM SpR
	I think people still feel empowered by being told and explained and you building a rapport and trust, I suppose.	GIM Consultant
	as long as people are prepared to be told the truth rather than just prepared to be deferred to, that’s fine.	GIM Consultant
	You can do a conversation badly. You can learn your framework and deliver a conversation well textbook, but that doesn’t necessarily make it land well. The landing well bit is in the communication. That’s the skill. That’s the real skill, is having the communication skills. Because you constantly modify what you say, based on what you get back.	Palliative Care Consultant
Conflict happens but should be avoided	I’ve seen it with clinicians who were of the same culture as a family who one would have perceived to be asking for treatments which really are not indicated and won’t work. So I think the cultural clash then is between a particular cultural family and more the British medical way.	ICM Consultant
	The big extraneous factor obviously, apart from the patient, is their family. I did once have an extremely hostile reaction to CPR. A gentlemen. . . I remember it so well	ICM SpR
	I’ve had other times when people have said, oh, you’re giving up on me, and I think, oh, that didn’t go well,	GIM Consultant
	I try to be explicit about the fact that it’s not a smorgasbord of options, take your pick, but without using the thing that gets thrown in so much, which is it’s a medical decision. Which I always think is a really bad way of starting the conversation by saying I’m going to talk to you and offer you an opinion and offer you an option, but actually it’s not an option. I’ve already decided what the answer is and you don’t get to decide, but I’m going to pretend as if you do get to decide. How is that conversation going to go? Would you like this? No, you can’t have it, I’m sorry. Here’s tonight’s menu and you can only have the fish.	GIM Consultant
	But I think I go down on the side of caution and talking about something more than not talking about it because otherwise, it’ll just come back to bite you later when somebody else comes along and suggests it. Our doctor never offered that to me.	Palliative Care Consultant
	I have that a lot on my mind because I see a lot of patients, and then the more patients you see, the more chance you’re going to end up in court.	EM SpR
	And if by not escalating, or not being seen to give what may well be a no-hope case everything you possibly can, in inverted commas, which may prolong the dying process by three or four days and be unpleasant for the patient, if not doing that causes significant distress for the relatives, and also increases the chance of another complaint coming your way, and the hospital legal team getting involved, and a datix that need investigation and the kind of professional reputational jeopardy you put yourself in with that, then I think you have to play the game.	EM Consultant

## Main themes

### Theme 1: An Unequal Partnership

#### Definition: The factors informing a TEP decision are so complex and unfamiliar that patients and clinicians are not a meaningful partnership to make decisions

Clinicians appeared confident and fluent synthesising large quantities of complex information. This was contrasted with their perception of patients’ relative unfamiliarity with future health planning and medical intervention.

##### Clinicians synthesise and interpret complex information amidst uncertainty

Clinicians exhibited what seemed a logical, nuanced, multifaceted thought process integrating the patient’s health state, multiple potential treatment modalities, anticipated sequelae of treatment and predicted survival and quality of life balanced with possible treatment trauma. TEP decisions involved uncertainty at many levels: limited information at the beginning of an acute admission; unknown circumstances of deterioration; impossibility of predicting accurately patient outcomes; subjectivity in defining ‘success’ and ‘futility’. It did not often seem possible to integrate patient values explicitly in such an ambiguous, complex initial deliberation.

TEP decisions seemed complex. Clinicians considered multiple individualised background factors, including comorbidities, functioning, frailty, age, admission frequency, exercise tolerance and disease trajectory, as well as the acute health condition. As demonstrated by this intensivist (full quotation in Table 1), there were detailed thought experiments predicting how a patient’s journey might proceed.

‘It’s never a single organ failure when the heart or the lungs are involved, they bring the other organs along with them. If I end up tubing him first, then I would not manage to extubate him because his dependence on the ventilation would be quite significant’. ICM_SpR1

However, ‘gut feeling’ appeared equally important in the face of this complexity and uncertainty, and sometimes, the factual basis for decision-making described above appeared a *post hoc* rationalisation for an instinctive decision. Whilst predictions were accepted to be fallible, treatments were deemed inappropriate on the basis that they ‘won’t work’ and ‘futility’ was frequently referenced, variably denoting survival, quality of life or appropriateness of a treatment choice for the condition. Some observed that the clinical picture could evolve and considered the value of a trial of treatment. Occasionally, proxies, such as use of a commode or Zimmer frame indicating frailty unlikely to benefit from aggressive escalation, could be used to shortcut a complex decision. This internal medicine physician reflected that outcomes from resuscitation and therefore the correct TEP decision often seemed intuitively clear.

‘The truth is yes, usually, you kind of know’. GIM_Consultant1

##### Balancing acceptable outcomes within the limitations of treatment possibilities is unfamiliar to the general public

Whilst clinicians considered TEP conversations part of their everyday practice, they observed that patients and families struggled with the unfamiliar conversation and had unrealistic expectations of medical intervention.

Clinicians commented that conversations about death seemed alien to patients and families, perhaps reflecting wider societal attitudes. It was valuable but unusual if a patient had already considered health deterioration, perhaps following previous healthcare encounters or TEP conversations. Media representations of resuscitation were considered inaccurate and unhelpful. Some felt that news coverage of intensive care and ventilators during COVID-19 had increased awareness of TEP, but, in general, patient understanding of treatment modalities was perceived to be limited. Some clinicians therefore found it challenging to broach TEP conversations because they could be greeted with shock and distress, especially in the acute setting where the TEP conversation usually followed a more welcome discussion about management of the acute presenting condition. This could negatively impact on doctor–patient relationships:

‘If they’ve never heard the word ‘resuscitation’ before, then I think it puts a barrier between us and the patient’. GIM_Consultant2

Clinicians felt that patients and families struggled to grasp the complexities and limitations of medical treatment. This was especially the case for acutely unwell patients with fluctuating capacity and preferences due to acute physiological factors as well as anxiety. Some clinicians reflected that professionals witness the worst outcomes and are therefore pessimistic. There was unresolved tension between clinician knowledge and patient expectation: clinicians appreciated patients’ fight for life, but thought it difficult to appreciate how bad outcomes can be without professional or personal experience. This could be particularly challenging when families demanded treatments that clinicians believed inappropriate.

‘Human nature is we fight to survive so being alive is better than not if you speak to someone on the street about it. So, they don’t really necessarily know what a life of being wheelchair bound or PEG fed or not being able to walk again or not being able to care for yourself and going into a care home is actually really like’. GIM_SpR2

Therefore, clinicians felt that patients could not often be partners in the decision-making process. Instead, clinicians had expectations of commonly preferred outcomes and sought to make and communicate decisions accordingly on a basis of trust, using a simplified rationale. The term ‘paternalism’ was mentioned several times, in this setting describing an authoritative approach to communicating with patients about TEPs. Generally, the term was implied to represent old-fashioned, flawed practice, contrasting with improved current approaches where patients are included in conversations to help them understand their condition and treatment. Occasionally, clinicians volunteered, as if expecting this to be controversial, that they ‘liked paternalism’ if it meant that they could use their expertise to make good clinical decisions.

### Theme 2: Options Without Equipoise

#### Definition: There are right and wrong TEP decisions, for which doctors take responsibility

The ‘gut feeling’ about likely clinical trajectory described in Theme 1 co-existed with what seemed to be a strongly-felt moral compass guiding clinicians’ actions. Together, these informed an instinctive sense of personal and professional responsibility for what were considered appropriate escalation strategies. For many, decisions involving more intensive treatment escalation for the frailest appeared unethical whilst it was presumed that patients deemed ‘good candidates’ (a term used for those patients anticipated to benefit) for escalation would not require discussion about TEP; it was unclear how precise thresholds for escalation were determined. There was variability in how much patients might be consulted in ambiguous decisions.

##### There are ethically appropriate decisions

Ethical principles were not explicitly articulated or balanced, but appeared implicit in clinicians’ reasoning about good decision-making.

Clinicians sought TEPs which increased chance of survival to an acceptable quality of life. They appreciated that predicting outcomes is uncertain and that experience of health is subjective, but it was considered reasonable to deliver a professional interpretation according to patients’ anticipated ‘best interests’. That said, there was variation in what quantitative and qualitative outcomes were considered acceptable. Some baseline health states, such as severe dementia, were considered less appropriate to prolong using invasive treatment. Personal instincts and experience of seeing patients suffer following treatment escalation meant that many felt it difficult to view some outcomes positively.

‘I think you’re always going to put your own idea on what’s a good quality of life. Having seen it and having seen patients come in, they’re so dependent, and I think then, yes’. GIM_Consultant2

Fear of doing harm through escalation appeared important. Decisions to limit treatment occurred when clinicians assessed that intervention would cause more harm than good. Clinicians were conscious that treatments could be aggressive and felt that inflicting them on some patients was unacceptable. Some remarked a tendency to overtreat in some medical specialties, or described a ‘slippery slope’ in the ICU towards more and more invasive treatment. Many described scenarios where decline towards death is inevitable and emphasised a duty to preserve dignity at end of life. Some also remarked clinicians’ roles in enacting treatment decisions and an instinctive aversion to delivering what was perceived as excessive intervention, such as intubating a very elderly patient.

‘There’s something very ugly about trying to provide…I mean... tube...a 92 year old gentleman, and chest compressions. It’s just…wrong’. ED_SpR1

Resource implications on appropriate treatment decisions were viewed variably and the discussion sometimes seemed uncomfortable. Some felt that resources such as ICU bed availability did influence decisions but that this was reasonable in a national health service; others stated that correct decisions were independent of resource and appropriate escalation would always be achieved if needed; and some felt uncomfortable that resources prevented some patients, such as those older and frailer, being given a chance at escalation and referenced stringent ceilings of care during the COVID-19 pandemic. Consultants also commented on the implications for staff morale of requiring teams to attempt resuscitation on patients where it was unlikely to be successful and the implications of distressing resuscitation scenarios on a fragile workforce.

‘There’s a junior doctor team and it’s traumatic for them and the nurses, and then it’s utilising resources poorly as well’. GIM_Consultant2

Clinicians emphasised that ‘everyone’s different’ but this was often realised as individualised decisions *for* rather than *with* patients. It was frequently remarked that patients must be able to refuse treatment, even if they cannot demand it, although there appeared a reticence to discuss TEP where full escalation was assumed. Refusal of recommended treatment could feel uncomfortable and lead to further discussion and rigorous assessment to confirm decision-making capacity, but the decision was respected. When clinicians had a neutral attitude to a TEP, some invited patients’ choices within a remit of options they believed appropriate. These conversations involved more detailed discussion, so that patients could embark on treatment ‘with their eyes open’ about possible outcomes.

‘And I guess, medically you have your boundaries about things before you go into the conversation, if that makes sense. So, you know what medically doesn’t make sense, and what you’re not putting on the table’. PalliativeMedicine_Consultant1

##### Doctors shoulder individual and professional responsibility for the TEP decision

Clinicians took TEP decisions seriously and appeared to feel ultimate weight of responsibility for making good decisions, both personally and as viewed by colleagues and society. The TEP decision appeared high stakes and achieving it a source of anxiety.

Clinicians considered TEPs part of their professional duty and believed their expertise was important in making difficult decisions on behalf of others. They emphasised the need to be realistic, weighing up harms and benefits of treatments and only trying to treat what is treatable. It was frequently mentioned that TEPs, and especially Do Not Attempt CardioPulmonary Resuscitation (DNACPR) decisions, are legally a ‘medical decision’, and patients cannot demand treatment. Several clinicians compared this with other areas of clinical decision-making or even decisions made by other professional groups.

‘It’s the same way that, sure, I can make a shared decision with my mortgage lender, but ultimately I don’t know what I’m doing, and they will be giving me the money’. GIM_Consultant3

Clinicians aspired to high standards and were aware of colleagues’ scrutiny. Some commented on others’ poor communication skills, for example, not framing TEP conversations within a broader context of a patient’s overall health, and others remarked on skills valued in their own specialty that they perceived to be lacking in others. Clinicians were aware of local norms and making a TEP decision outside of the status quo felt uncomfortable.

‘It does still reflect on you and you will still get blamed as looking after that patient if that patient is resuscitated, and then intubated. And the ITU team look at the patient and the comorbidity state, and go, this patient actually shouldn’t have been resuscitated. So, you'll still have to answer for that decision’. GIM_Consultant4

Instinctive practice on appropriate TEPs was considered to reflect general colleague consensus about most decisions, such as DNACPR for a very frail patient or full escalation for a fit one. Colleagues might be consulted to advise in complex cases, but this was not deemed necessary for most decisions. That said, it was considered inevitable that different clinicians ‘leaned’ towards more or less intervention. This was informed by personal beliefs and clinical experiences of moral distress or learning from previous cases. Increased experience brought confidence with making good decisions. One clinician described seeking their own ‘medical truth’ for a decision. It was also remarked that treatment thresholds varied between centres.

### Theme 3: Decisions with shared understanding

#### Definition: The TEP conversation should ideally achieve patient involvement, understanding and agreement

Having formulated a TEP according to the medical complexity and moral compass described in Themes 1 and 2, clinicians generally sought understanding and agreement from patients. Patient or family disagreement could derail a ‘good’ TEP. Therefore, clinicians sought to retain tight control over the high-stakes discussion rather than ‘sharing’ the decision.

##### Achieving patient understanding and agreement is important

Clinicians considered communication an important responsibility during the TEP process. They sought patient understanding of the decision and agreement with, or at least acceptance of, the medically proposed plan. Some commented that if patients are to be included in the conversation, they had to be willing to be told a hard truth.

The TEP conversation was appreciated to be skilled and nuanced, requiring individualised communication. Some registrars felt that they lacked training in the complex conversation. Consultants reflected that communication skills are built with experience. Doctors from some groups, such as emergency medicine, considered detailed TEP conversations better conducted by those with relevant skills, time and personality. Clinicians emphasised the importance of ensuring patients were informed of the TEP and described approaches such as avoiding medical jargon. They were aware of a legal duty to inform about CPR decisions. Some felt that patient understanding meant they were empowered and had the opportunity to refuse treatments, but mostly, it simply seemed the right thing to do.

‘You can do a conversation badly. You can learn your framework and deliver a conversation well textbook, but that doesn’t necessarily make it land well. The landing well bit is in the communication. That’s the skill’. PalliativeCare_Consultant1

The clinician usually embarked on a TEP conversation knowing the preferred outcome and used various techniques to gain agreement. These included graphic descriptions of aggressive resuscitation, reassurance that the moral weight of the decision lay with the clinician rather than the patient or family, explanation of medical reasoning, ‘planting a seed’ as a warning shot and revisiting the conversation until an agreement was secured. Clinicians felt that their demeanour contributed to the conversation’s success, where trust increased the chance of agreement, and some reassured patients that they would give the same advice to their own families. Goals of care could be used to convince patients of a need for treatment limitation, to avoid a feared outcome. Many had a tried-and-tested narrative. Clinicians described being more paternalistic if they felt a patient view was wrong. Agreement with the proposed plan was sometimes accepted even if the patient did not appreciate the full details. Some clinicians remarked that SDM was good as long as the decision was that chosen by the clinician.

‘It is a shared decision as long as we reach the decision that we want I guess is my thought about that because I think we may end up with a lot of people saying I want to be for everything and that isn’t right based on their presentation’. GIM_SpR1

##### Conflict happens but should be avoided

Conflict was perceived to be prevalent around TEP discussions. It was considered challenging and distressing. Clinicians sought, at best, to manage or avoid conflict or, at worst, to limit damage to their own professional lives.

Clinicians hoped to maintain good patient relationships and recognised that this could be threatened by the TEP conversation, which was considered by definition a difficult conversation because it anticipated deterioration and potential death. Several clinicians described memories of challenging discussions. In particular, conflict arose when a decision to limit treatment was communicated to families. Clinicians described the challenge of differentiating between patient and family views. Sometimes, conflict centred around cultural beliefs, where a medical plan to limit escalation was disputed. Clinicians found this difficult, torn between desire to respect individuality and respect different cultures and a strong sense that escalation was inappropriate according to their medical training.

‘I've seen it with clinicians who were of the same culture as a family who one would have perceived to be asking for treatments which really are not indicated and won't work. So I think the cultural clash then is between a particular cultural family and more the British medical way’. ICM_Consultant1

Clinicians nonetheless strived to avoid conflict where possible. Communication breakdown and lack of consistency between explanations were seen to precipitate disputes. Anticipating patient upset, clinicians stressed that the TEP conversation was routine and reassured patients that deterioration was unlikely and the aim was to prevent it. It was recognised that many patients and some clinicians perceive a DNACPR decision to result in limitations to other treatments, and clinicians emphasised that not escalating did not mean not caring. Timing the conversation was important, as embarking before a trusting relationship was established could lead to problems. Some were paternalistic to gain agreement but others found that this could increase conflict.

‘I try to be explicit about the fact that it’s not a smorgasbord of options, take your pick, but without using the thing that gets thrown in so much, which is ‘it’s a medical decision’. Which I always think is a really bad way of starting the conversation by saying I’m going to talk to you and offer you an opinion and offer you an option, but actually it’s not an option’. GIM_Consultant1

Sometimes, clinicians conceded to patient or family requests for further escalation. Some considered compromise an acceptable outcome reflecting value of patient autonomy or kindness to the family who once grieving would have the comfort that everything was tried. Others reflected that it was not worth the argument, especially where conflict could lead to extreme clinician stress and an unsuccessful outcome was inevitable regardless of treatment escalation.

Clinicians described behaviour informed by anticipation of formal complaints and legal repercussions. Second opinions were considered helpful in case a decision was questioned later. Rather than overall patient goals of treatment, clinicians felt duty-bound to discuss treatments in order to document unambiguously what was agreed in case of future scrutiny. Documentation was key, partly as a means of communicating with the team but also in case of legal action.

‘If not doing that (escalating) causes significant distress for the relatives, and also increases the chance of another complaint coming your way, and the hospital legal team getting involved, and a datix^1^ that needs investigation, and the kind of professional reputational jeopardy you put yourself in with that, then I think you have to play the game’. EM_Consultant1

## Discussion

This is the first study of which we are aware specifically exploring clinician perspectives on SDM around TEP in the acute medical setting for older adults with capacity to discuss treatment escalation [1]. This study suggests that SDM fully incorporating patient preferences with clinical opinion is seldom a priority for clinicians considering TEP in the acute medical setting. Lack of perceived equipoise together with clinical complexity and uncertainty motivates clinicians to develop a TEP they consider medically appropriate, about which shared patient understanding is important.

This study sits within a field of research examining conversations planning for deterioration in the acute general hospital setting, including CPR decision-making [39, 40] the ‘Recommended Summary Plan for Emergency Care and Treatment’ intervention evaluation [41–45] and referral to intensive care [46, 47]. It is also important to note relevant legal cases, including Tracey [48], Winspear [49] and Montgomery [29]. This research contributes by exploring specifically, from a clinician perspective, ‘shared’ rather than wider decision-making and TEP beyond CPR decisions.

Complex decisions were a barrier to involving patients. Previous research has described the complexity of TEP decisions, use of heuristics to navigate decision-making in uncertainty [43, 45, 46, 50] and perception that patients lack sufficient understanding [51, 52]. Even with experience of treatment escalation, patients may not be able to conceptualise thresholds for future acceptable treatment outcomes [53]. However, patients, especially older people [54], or those with less pre-existing knowledge [55], may not feel empowered to contribute to decision-making [56] or fear distressing conversations [55]. Clinicians do not always find effective strategies to communicate medical ideas or feel able to involve patients [44, 46, 57]. The extent to which clinical decision-making complexity and nuanced appreciation of outcomes can be made accessible to patients is not clear.

Clinicians took responsibility for making what they felt to be ethical decisions. Other studies concur that clinicians consider TEP a ‘medical decision’ [41, 43–45, 50, 58–61], making difficult decisions [59] in line with their clinical view [62]. SDM is an ‘ethical imperative’ [2], especially in society prioritising ‘autonomy’ [63], to align treatment with patient goals [64]. However, clinicians also have duties of ‘beneficence’, ‘nonmaleficence’ and ‘justice’ as established in the pillars of biomedical ethics [65], and there is much discussion regarding how the principles can conflict [66]. In TEP decision-making, patient requests for ‘inappropriate’ treatment [67] are considered challenging when clinicians and patient have differing perspectives on acceptable quality of life [68], clinicians feel that patients do not appreciate severity of potential suffering [69], or there is resource consumption in a healthcare system with finite resources [70, 71]. Clinicians appeared practised in reconciling numerous influences to reach a resolution, whilst what constitutes a ‘good’ decision remains undefined.

There was fear of conflict and legal repercussions and use of strategies to manage TEP decisions to a medically acceptable conclusion. Awareness of tensions is prevalent in many studies exploring TEP conversations. Whilst clinicians are often committed to resolving difficult conversations and securing a decision, TEP conversations appear to be a challenging aspect of daily practice, provoking clinician as well as patient upset and damaging rapport [41, 43–45, 50, 57–61, 72, 73]. Nonetheless, there is a legal requirement to inform patients with capacity about CPR decisions [24, 48]. Clinicians therefore seek to navigate persuasive conversations towards an outcome they deem acceptable whilst maintaining relationships [43, 57, 74].

## Implications

This study was conceived as an open exploration of clinicians’ perspectives rather than measurement of ‘good’ practice according to existing guidance. The notion of SDM as ideal practice is socially informed, and therefore its realisation in clinical environments warrants continual evaluation. Nonetheless, it is interesting to note that current UK guidelines on CPR decisions expect SDM wherever there is a ‘realistic’ chance of CPR ‘success’ [24, 27]. Our study suggests that clinicians do not perceive scope for SDM as often as this implies. Clinicians assessed, according to quantitative or qualitative estimates, what degree of treatment escalation would be appropriate. For patients anticipated to do well following escalation, it was considered unnecessary to discuss TEP (although the patient’s right to refuse treatment was important); for those where treatment limitation was deemed appropriate, shared understanding was sought; for the minority, where ‘success’ was possible but equivocal, SDM was considered within medically acceptable confines.

Therefore, we suggest that existing aspirations for SDM in TEP and clinician perspectives do not align. There are a number of possible initiatives, based on our thematic analysis, by which practice might move closer to expectation. From Theme 1, public education around limitations of medical intervention could narrow the gap between patient and clinician understanding. From Theme 2, clinician education from early stages of training could encourage a broader view of ‘acceptable’ outcomes where patient values are prominent. Acknowledging tensions across the wider health service, addressing resource limitations could conceivably alter approaches to treatment limitation. From Theme 3, communication skills training might increase confidence in navigating challenging conversations. However, an alternative view is that current expectations of SDM in TEP are unrealistic. The clinicians in this study demonstrated enormous commitment to achieving what they considered best outcomes for their patients. From a position of considerable collective experience and multispecialty perspective, they did not see SDM to be widely applicable across patient cases and to the broadest degree of sharing. We cannot propose a solution, but it seems important to outline this paradox as a foundation for iterative debate towards practicable guidelines that can satisfy different stakeholders.

### Limitations

This study was conducted at one NHS Trust, which may limit transferability. However, standards governing TEP decision-making are established UK-wide. Doctors frequently move geographically as part of their training, and their views are likely to reflect the whole scope of their clinical experience having worked in different organisations.

In keeping with qualitative methodology, the sample size was ultimately determined by data saturation and analysis focussed on depth rather than breadth. Through purposive sampling of specialty and clinical seniority, we sought to capture a range of viewpoints.

In keeping with the reflexive thematic analysis approach, interviews and coding were performed by one researcher. There was iterative discussion with the wider research team and engagement with reflexivity to maximise validity.

Clinicians’ reflections based on vignettes may not represent their actual practice. Using a constructivist approach, we have considered meaning and knowledge as socially produced [37], wherein clinicians actively interpret sociolegal, personal and professional contexts to construct meanings from their clinical experience and communicate a perspective on the role of SDM in TEP.

## Conclusion

This qualitative interview study sought to understand clinician perspectives on Shared Decision-Making in Treatment Escalation Planning. *Sharing* decision-making by incorporating patient preferences with clinical opinion was seldom a priority. Clinicians took responsibility for instinctive, ethically driven decision-making synthesising complex information and sought shared understanding with patients. This research has implications for health policy and education. We propose a need for debate amongst stakeholders to define the role of Shared Decision-Making in Treatment Escalation Planning with older patients in the acute medical setting.

## Supplementary Material

aa-24-0008-File002_afae204

## Footnotes

1 An incident reporting system used in the NHS
