# Supplementary material for: Shared decision-making with older people on TReatment Escalation planning for Acute deterioration in the emergency Medical Setting: a qualitative study of Clinicians’ perspectives (STREAMS-C)
Source: Age Ageing. 2024 Sep 25;53(9):afae204. doi: 10.1093/ageing/afae204 (PMC11424886; doi:10.1093/ageing/afae204)
Supplement: aa-24-0008-File002_afae204 [file aa-24-0008-file002_afae204.docx]

APPENDIX

**Shared decision making with older people on TReatment Escalation planning for Acute deterioration in the emergency Medical Setting: a qualitative study of Clinicians’ perspectives (STREAMS-C)**

Table of Contents

[APPENDIX 1: Interviews and vignettes 1](#_Toc153818267)

[APPENDIX 2: Detailed description of reflexive thematic analysis approach 2](#_Toc153818268)

[APPENDIX 3: Consolidated criteria for reporting qualitative research 2](#_Toc153818269)

[APPENDIX 4: Findings in context 4](#_Toc153818270)

## **APPENDIX 1: Topic guides and vignettes**

Topic guide questions were developed through iterative discussion across the whole team. They were informed theoretically by models of the Doctor Patient Relationship [1-5]. While there exist UK guidelines on CPR decision-making [6], these were not explicitly referenced during topic guide drafting as this was designed to be an open exploration of views rather than audit of ‘good’ practice. Clarifying questions added during the first four interviews are shown in italics. These questions were prompted by concepts mentioned by participants which resonated with the wider TEP and SDM literature and seemed important to explore in more detail, or were considered helpful to add structure and focus the interview on the research question.

Participants were asked to describe their approach to TEP, invited to discuss the vignettes and finally directly asked their views on SDM in TEP.

The three vignettes, developed by clinical members of the research team, described a hypothetical capacitous patient with multiple comorbidities presenting with a urinary tract infection. He was being admitted under the acute medicine team but not acutely deteriorating. Across the three scenarios, age, severity of comorbidity and functional dependence increased. It was made clear that, as with many older people in the acute setting, he had multiple interacting chronic health conditions rather than primarily a single organ failure. Participants were probed to consider their responses to different reactions from the patient, for example declining treatment they were prepared to offer or demanding treatment they considered inappropriate.

**INITIAL TOPIC GUIDE**

- Explore clinicians’ approach to treatment escalation planning conversations with a focus on the perceived role of shared decision making
- Consider whether the treatment escalation planning conversation or decision vary with regards to shared decision making depending on patient clinical factors

Thank you for agreeing to participate in this study. Today I’d like to find out how you think about decision making around treatment escalation planning for patients admitted to hospital on the acute medical take.

I have some scenarios to help set the scene and some questions to make sure I’ve covered everything but really this should just be a conversation.

This should take no more than one hour. I will be recording our conversation so that I’m free to listen to you.

Everything you say will be confidential and anonymised for the study. Do you have any questions? Are you happy to proceed?

1. What is your understanding of Treatment Escalation Planning (/respect form/ceiling of care decisions)?

What does it mean to you (in the acute medical setting)?

Is it useful?

When are you involved in TEP, how do you see your role?

*Has your approach changed over time, if so why?*

1. How would you approach Treatment Escalation Planning for each of these patients?

You have been given three scenarios. Please read them now.

I’d like you to imagine you are making a treatment escalation plan for each of these patients.

>Can you talk me through step by step how you would approach it?

*- what is an ‘acceptable’ outcome, how do you decide a threshold, how bad is too bad?*

*- how/why does your view change across the three scenarios?*

*- Is there a ‘right’ decision?*

*- what is ‘futility’?*

*- would it make a difference if there were unlimited resources?*

>What sort of conversation would you have with the patient?

*- do you ever talk about shared goals of care?*

Are you more likely to think/talk about TEP for some patients than others?

1. Involving patients and families in Treatment Escalation Plans in general?

What do you think about involving them?

*- where are you on a spectrum from giving information and leaving patients to make a decision, recommending a decision, communicating your decision*

How/when would you involve them – can you talk me through?

>Would you explain the risks, benefits, alternatives of your proposed TEP?

What if there is disagreement?

*- how much is litigation a concern?*

What would make you think the conversation had gone well?

What do you think about shared decision making in treatment escalation planning?

*What is the most important thing to you in this subject?*

Scenario A

66M with stable angina, type 2 diabetes mellitus (on metformin), asthma (never admitted with it), current smoker (10 pack year history), no alcohol. Fully independent, able to walk around the park unaided outside. Lives with wife in house. No admissions in the last year.

He has been admitted with a urinary tract infection and is currently stable.

He has capacity to make decisions about treatment escalation. It is hospital policy that patients admitted under acute medicine have a documented treatment escalation plan.

Scenario B

79M with ischaemic heart disease (coronary artery bypass graft 3 years ago), atrial fibrillation (on direct oral anticoagulant), type 2 diabetes mellitus (on insulin), asthma (never admitted with it), diverticular disease, ex-smoker (20 pack year history), no alcohol. Has once a day package of care and can mobilise short distances approx. 15m outside with stick, limited by breathlessness; lives in ground floor flat so never has to do stairs. Can get dressed independently but wife waits nearby. One admission in the last year with pneumonia.

He has been admitted with a urinary tract infection and is currently stable.

He has capacity to make decisions about treatment escalation. It is hospital policy that patients admitted under acute medicine have a documented treatment escalation plan.

Scenario C

92M with ischaemic heart disease (coronary artery bypass 16 years ago) and aortic valve replacement, atrial fibrillation (on direct oral anticoagulant), type 2 diabetes mellitus (on insulin), asthma (never admitted with it), diverticular disease, osteoarthritis, hearing impairment (has hearing aids), ex-smoker (20 pack year history), no alcohol. Has a four times a day package of care. He can mobilise to the commode with a Zimmer frame but cannot do stairs. Lives alone. Needs help washing and dressing, can toilet independently. Two admissions in the last year with pneumonia.

He has been admitted with a urinary tract infection and is currently stable.

He has capacity to make decisions about treatment escalation. It is hospital policy that patients admitted under acute medicine have a documented treatment escalation plan.

## **APPENDIX 2: Detailed description of reflexive thematic analysis approach**

Reflexive thematic analysis was performed with reference to Braun and Clark.[7]

First, BEW familiarised herself with the data through rereading transcripts and listening to interview audio recordings.

Next, BEW systematically applied line-by-line coding to the entire transcript dataset; semantic and latent codes were used to capture explicit and implicit meaning. An inductive approach was used to remain open to new perspectives in the data rather than seeking to understand existing theory. This was considered important to avoid judgement of clinicians’ views as ‘good’ or ‘bad’ according to existing TEP guidelines and SDM literature. BEW was aware that her clinical experience could prompt premature meaning-making informed by her own perspectives, so a large number of codes (1943 codes) were applied initially in an effort to capture granularity in the data. All codes were reviewed with the transcripts to ensure that each code was distinct and made sense in isolation from the data.

Mindful that this fine-grained approach, while important to achieve rigor and some separation from the data, would be difficult to assimilate, codes were then grouped according to shared descriptive meaning. Following a period of reflection and wider research team discussion, initial candidate themes were generated, and coding groups arranged into these themes. At this stage, the original transcripts were revisited in full to ensure that the themes captured meaning from across the dataset. Individual quotations were extracted to illustrate themes, with care taken to ensure that these quotations reflected meaning in context of the whole interview. Themes were refined in an iterative process, involving discussion with all the authors.

The writing process prompted further discussion within the research team and revision of the themes to ensure that each one had a central organising concept without overlap between themes. The themes were defined and named. Subthemes were developed after theme generation to capture clinically-relevant facets of the central organising concept. Coding was supported by computer assisted qualitative data analysis software NVivo (NVivo qualitative data analysis software, QSR International Pty Ltd, release 14.23.0) but theme generation was performed manually.

## **APPENDIX 3: Consolidated criteria for reporting qualitative research**

| No | Item | Guide questions/description |  |  |
| --- | --- | --- | --- | --- |
| Domain 1: Research team and reflexivity |  |  |  |  |
| Personal Characteristics |  |  |  |  |
| 1. | Interviewer/facilitator | Which author/s conducted the interview or focus group?  BW |  |  |
| 2. | Credentials | What were the researcher's credentials? *E.g. PhD, MD*  MBChB, MRCP |  |  |
| 3. | Occupation | What was their occupation at the time of the study?  Medical registrar doctor, PhD student |  |  |
| 4. | Gender | Was the researcher male or female?  Female |  |  |
| 5. | Experience and training | What experience or training did the researcher have?  Previous experience as first author of qualitative methods study, Attendance at multiple courses including King’s College London ‘Introduction to Qualitative Research’ 2022, European Association for Communication in Healthcare Summer School 2023, University College London ‘Writing and Publishing Qualitative Research’ 2023 |  |  |
| Relationship with participants |  | BW and SB are current or former colleagues of some of the participants. This is reported in the ‘Reflexivity’ section |  |  |
| 6. | Relationship established | Was a relationship established prior to study commencement?  BW and/or SB have pre-existing professional relationships with approximately half of the participants. There was no previous relationship with other participants. |  |  |
| 7. | Participant knowledge of the interviewer | What did the participants know about the researcher? e*.g. personal goals, reasons for doing the research*  All participants were supplied with a participant information sheet outlining the research study and explaining that the interviewer BW is a PhD researcher and her academic area of interest |  |  |
| 8. | Interviewer characteristics | What characteristics were reported about the interviewer/facilitator? e.g. *Bias, assumptions, reasons and interests in the research topic*  The interviewer’s profession and level of clinical seniority are reported in the ‘Reflexivity’ section |  |  |
| Domain 2: study design |  |  |  |  |
| Theoretical framework |  |  |  |  |
| 9. | Methodological orientation and Theory | What methodological orientation was stated to underpin the study? *e.g. grounded theory, discourse analysis, ethnography, phenomenology, content analysis*  Reflexive Thematic Analysis |  |  |
| Participant selection |  |  |  |  |
| 10. | Sampling | How were participants selected? *e.g. purposive, convenience, consecutive, snowball*  Purposive, described in the ‘Study Design’ section of the Methods |  |  |
| 11. | Method of approach | How were participants approached? e*.g. face-to-face, telephone, mail, email*  Face to Face and email |  |  |
| 12. | Sample size | How many participants were in the study?  26 |  |  |
| 13. | Non-participation | How many people refused to participate or dropped out? Reasons?  An email invitation was sent to ‘mailing lists’, it is not possible to establish how many people refused to participate or why. However, no one refused directly or dropped out. |  |  |
| Setting |  |  |  |  |
| 14. | Setting of data collection | Where was the data collected? e*.g. home, clinic, workplace*  In person at the NHS Trust in the participants’ offices or via video conferencing with the interviewer at home and the participant at home or in their NHS office |  |  |
| 15. | Presence of non-participants | Was anyone else present besides the participants and researchers?  No |  |  |
| 16. | Description of sample | What are the important characteristics of the sample? *e.g. demographic data, date*  Medical specialty, level of clinical seniority and gender are recorded. |  |  |
| Data collection |  |  |  |  |
| 17. | Interview guide | Were questions, prompts, guides provided by the authors? Was it pilot tested?  The interviews were semi-structured, meaning that the interviewer supplied verbal prompts according to the topic guide but the conversation was free-ranging. The interview questions were refined over the first four interviews. Vignettes of hypothetical clinical cases, designed by clinical members of the research team, were supplied as written information, read real-time during the interview by participants, to prompt discussion. |  |  |
| 18. | Repeat interviews | Were repeat interviews carried out? If yes, how many?  No |  |  |
| 19. | Audio/visual recording | Did the research use audio or visual recording to collect the data?  Audio recording |  |  |
| 20. | Field notes | Were field notes made during and/or after the interview or focus group?  Reflexive field notes were recorded immediately after each interview |  |  |
| 21. | Duration | What was the duration of the interviews or focus group?  Interviews lasted on average 61 minutes, range 35 to 79 minutes |  |  |
| 22. | Data saturation | Was data saturation discussed?  Recruitment continued until data saturation was reached, defined as the interviewer hearing no new ideas during the interviews. This is described in the ‘Study design’ section of the methods |  |  |
| 23. | Transcripts returned | Were transcripts returned to participants for comment and/or correction?  No |  |  |
| Domain 3: analysis and findingsz |  |  |  |  |
| Data analysis |  |  |  |  |
| 24. | Number of data coders | How many data coders coded the data?  One – BW – in keeping with reflexive thematic analysis methods. However, themes were generated through iterative discussion across the whole research team |  |  |
| 25. | Description of the coding tree | Did authors provide a description of the coding tree?  A formal coding tree was not used, in keeping with reflexive thematic analysis methods, but codes were clustered as part of candidate theme generation and rearranged iteratively as themes were reviewed in discussion with the team. |  |  |
| 26. | Derivation of themes | Were themes identified in advance or derived from the data?  Inductive coding was performed |  |  |
| 27. | Software | What software, if applicable, was used to manage the data?  Coding was supported by computer assisted qualitative data analysis software NVivo (NVivo qualitative data analysis software, QSR International Pty Ltd, release 14.23.0) and theme generation was performed manually. |  |  |
| 28. | Participant checking | Did participants provide feedback on the findings?  No |  |  |
| Reporting |  |  |  |  |
| 29. | Quotations presented | Were participant quotations presented to illustrate the themes / findings? Was each quotation identified? e*.g. participant number*  Yes |  |  |
| 30. | Data and findings consistent | Was there consistency between the data presented and the findings?  Yes |  |  |
| 31. | Clarity of major themes | Were major themes clearly presented in the findings?  Yes |  |  |
| 32. | Clarity of minor themes | Is there a description of diverse cases or discussion of minor themes?  Yes |  |  |

## **APPENDIX 4: Findings in context**

The TEP conversation was familiar to clinicians as an important, high stakes decision taking place in a pressurised clinical environment. The term Shared Decision-Making (between patient and clinician) was not familiar to all. Family involvement was deemed important, especially if there were questions about a patient’s decision-making capacity.

TEP was understood to mean levels of organ support, admission to the intensive care unit and whether CPR should be performed. There was debate on the value of integrating the CPR decision into a wider TEP discussion or acutely focussing only on CPR. Clinicians used their assessment of likely escalation outcomes to inform whether a TEP conversation should be initiated: it seemed particularly important to discuss with patients who were predicted to do badly and less important, or even problematic and impractical, to discuss with people who were ‘obviously’ for full escalation. It was considered too nebulous to discuss all eventualities and treatment options and many prioritised treatments and outcomes seeming most pertinent to the comorbidities and acute health problem. While discussions often focussed on limiting inappropriate intervention, some recalled advocating for more escalation, or argued that ‘invasive’ treatments such as arterial lines could be a kindness to avoid multiple distressing less invasive interventions such as venepuncture.

Timing the conversation appeared important. Preferences ranged from ‘cold light of day’ in the community with a familiar clinician, to as soon as possible into an acute admission establishing early clarity for both patients and the wider team, to a least ambiguous scenario once the deterioration had occurred. There was pressure to achieve a complete decision, encouraged by wider organisational guidelines and electronic records which nudged towards complete decision-making. Some commented that good TEPs need time, which was not always available in a busy acute setting with a ‘system in crisis mode’. A complete TEP was needed in case of deterioration during a fraught out of hours shift where embarking on further difficult conversations or enacting escalation could be more challenging: clinicians felt a responsibility to colleagues who would be left with their unfinished plan, and while some felt that this accelerated the decision unnecessarily, others believed it was an important and necessary part of their role.

In a descriptive analysis of clinicians’ responses to the vignettes, most felt that the least frail patient, whom they deemed ‘obviously’ appropriate for full escalation, did not require a TEP discussion, although some said that they would discuss if mandated by local policy. For the most frail patient, the majority were clear that a ‘ward based ceiling’ (no organ support) was most appropriate but almost half considered circulatory support (the perceived value of inotropes altering outcomes was debated) and some non-invasive respiratory support. The scenario of intermediate frailty and comorbidity received the most varied response and clinicians more frequently volunteered need for a patient involvement in the decision. Proposed TEPs ranged from ward-based care to single organ support to intubation to CPR.

1. Emanuel EJ and Emanuel LL. Four models of the physician-patient relationship. *Jama* 1992; 267: 2221-2226.

2. Parsons T. Epilogue. In: Gallagher E (ed) *The Doctor-Patient Relationship in the Changing Health Scene*. USA: Department of Health, Education and Welfare, Public Health Service, National Institutes of Health, 1976.

3. Veatch RM. Models for Ethical Medicine in a Revolutionary Age. What physician-patient roles foster the most ethical relationship? *Hastings Center Report* 1972; 2: 5-7.

4. Szasz T, Knoff W and Hollender M. The doctor-patient relationship and its historical context. . *Am J Psychiatry* 1958; 115: 522-528.

5. Kon AA. The Shared Decision-Making Continuum. *JAMA* 2010; 304: 903-904. DOI: 10.1001/jama.2010.1208.

6. British Medical Association RCU, Royal College of Nursing,. Decisions relating to cardiopulmonary resuscitation. Guidance from the British Medical Association, the Resuscitation Council (UK) and the Royal College of Nursing (previously known as the ‘Joint Statement’), <https://www.resus.org.uk/sites/default/files/2020-05/20160123%20Decisions%20Relating%20to%20CPR%20-%202016.pdf> (2016, accessed 02/20/2023).

7. Braun V and Clarke V. *Thematic Analysis: A Practical Guide*. SAGE, 2022.
